# Serving to win: exploring serve-reception effectiveness in high-level male and female sitting volleyball players

**DOI:** 10.3389/fspor.2024.1471094

**Published:** 2024-12-06

**Authors:** Valentina Cavedon, Marco Sandri, Paola Zuccolotto, Caterina Biasiolo, Carlo Zancanaro, Chiara Milanese

**Affiliations:** ^1^ Department of Neurosciences, Biomedicine and Movement Sciences, University of Verona, Verona, Italy; ^2^ BODaI-Lab, University of Brescia, Brescia, Italy; ^3^ Department of Diagnostics and Public Health, University of Verona, Verona, Italy

**Keywords:** performance analysis in sport, paralympic sport, adapted sport, match analysis, mixed-effects models

## Abstract

**Introduction:**

This study was planned to assess the association between serve efficacy and match outcome, and to investigate which factors are associated with serve efficacy in high-level male and female Sitting Volleyball players.

**Methods:**

The study sample was comprised of a total of 3,664 serving actions, performed during the 2020 Paralympic Games and the 2022 Sitting Volleyball World Championship. For each serving action, we considered serve efficacy (i.e., Point lost, Negative Serve, Positive serve and Point won), serve zone, type of serve technique, target zone of the serve, type of reception, receiving player and match outcome (i.e., match won, or match lost).

**Results:**

The Mixed-effects logistic regression model showed that serve efficacy is significantly associated with the match outcome, increasing serve efficacy being associated with increased chances of winning the game. The ratio between the probability of winning and losing the game started at 0.86 for a Point lost and increased to 1.67 for a Point won. Compared to Negative serves, Positive serves were associated with a higher probability that the opponent team would use a low reception rather than a high reception. In turn, a low reception was associated with a higher chance (from 45.1% to 58.3%) of the receiving team committing an error in the side-out phase.

**Discussion:**

Based on these results, it is recommended that high-level coaches focus on improving their players' serve efficacy by instructing them on managing risk, avoiding serves to the front zone, and reducing the likelihood of overhead receptions.

## 1 Introduction

Sitting Volleyball (SV) is a popular and fast-growing Paralympic team sport played worldwide at a competitive level by male and female athletes under the jurisdiction of World ParaVolley. SV is an attractive example of an accessible and inclusive sport where players with varying types and severities of impairments (e.g., upper/lower limb amputations, limb deficiencies, leg length differences, hypertonia, impaired muscle power, impaired passive range of movement, multiple sclerosis, ataxia, and athetosis) participate together (1–3). SV retains most of the major rules of standing volleyball (e.g., ball size, number of players on the court, strokes, scoring system), but it is played in a seated position on a 10 × 6 meter court with the net placed at a height of 1.15 meters for men and 1.05 meters for women (3).

Understanding performance is a major issue for coaches and technical staff, with performance analysis playing an important role in advancing the scientific understanding of a specific sport (4). In particular, in standing team sports it has been acknowledged that understanding how the skill performance indicators relate to scoring of points is useful for athletes and coaches (5). In standing volleyball, it has been underlined that it is important to know which skills better contribute to match success in order to promote an effective tactical orientation as well as for the design and development of training programs aimed improving the skills that provide a clear advantage to the team in terms of chance to win the match (6–8). In the last decades, a lot of performance analysis studies have been carried out focusing on standing volleyball and aiming at investigating the association between some key variables and an increased chance of winning matches (9–13).

In standing volleyball the serve and the serve-reception (referred to as “reception” from this point on) have been identified as two important predictors of team success (8, 12, 14, 15). Specifically, the importance of serving as a skill that can determine the outcome of the game is emphasized, as the course of action often depends on its efficacy (16–18). Serve-related factors influence on the reception efficacy (15, 19), and, in turn, the number of reception errors is also a predictor of winning or losing the match (8).

Depending on game tactics, the opponent, and the individual characteristics of the player, the primary goal of the serve in a given rotation, in both men's and women's volleyball, is to maximize its efficacy (18). Players typically aim to either score a direct point or disrupt the opponent's ability to construct an effective attack (8, 20), which includes reducing first-tempo attacks and enhancing block performance (21). This allows the serving team to better anticipate the setter's distribution and organize their block-defense strategy more effectively (22). In standing volleyball, research has also shown that a high level of serve efficacy is linked to factors such as serve type, reception zone, and the receiving player (23), with some gender-based differences observed (24). For instance, to enhance serve efficacy, male players tend to perform significantly more dynamic serves while jumping compared to female players. Additionally, men exhibit a lower ratio of aces to errors, suggesting that they are more willing to take risks in the serving phase (24).

As far as SV is concerned, no data are present in the literature dealing with the performance factors associated with the game outcome; to date, it has only been argued that the serve is a crucial factor for match success (25) but this hypothesis has never been investigated by the scientific community. The lack of a scientific understanding of the SV game means that coaches can only rely on their personal experience to support their tactical choices during the game and to plan training priorities. Furthermore, this can lead to the potential pitfall of automatically transferring research from standing volley to SV. Accordingly, there is a need to provide coaches and technical staff with more ecological knowledge by analysing the SV game from a scientific point of view.

When observing the SV game and comparing it to standing volleyball, it is evident that the game of volleyball, even when played in a sitting position, is characterized by an integrated playing action consisting of six fundamental skills: serve, reception, set, attack, block and defense. One of the main differences between standing volleyball and SV is that in SV players can attack or block their opponents' serve. Another noticeable difference is that all the fundamental skills of the game are performed while sitting directly on the playing court without the possibility of lifting the buttocks from the floor or jumping while hitting the ball (3). When considering the similarities between standing volleyball and SV, it is important to note that in SV, the serve is also the first offensive technical-tactical action, representing the start of every point. As in standing volleyball, the serve in SV is performed from the designated area and involves hitting the ball with the hand to direct it over the net and into the opponent's court. Additionally, like in standing volleyball, the serve in SV is the only closed skill in the game, allowing the player full control over the outcome, unaffected by preceding actions. This enables the player to decide the type of serve, the target zone, the ball's trajectory, and the force to apply. As far as the reception is concerned, also in SV this stroke is the first contact of the ball by the receiving team after the serve. Furthermore, as it happens when playing volleyball in a standing position, the SV reception is an intermediary linking open skill action that is influenced by the previous one (the serve) and that, in turn, influences the following actions.

While recognizing the significance of information from previous research on standing volleyball and noting a lack of studies focusing on the analysis of performance factors associated with match success in SV, we planned this exploratory research to analyse performance in SV. As a first step toward understanding which game-related SV skills better predict winning and losing in SV, we decided to investigate the serve and reception performance in high-level SV from the perspective of the serving team. In particular, this study aimed to assess the association between serve efficacy and match outcome, as well as to investigate which factors are associated with serve efficacy in SV. Based on the similarities between standing volleyball and SV we assumed that an increased serve efficacy could negatively affect the quality of the opponent's reception thereby increasing the serving team's chances of winning the game.

## 2 Materials and methods

### 2.1 Sample

**Figure 1:**
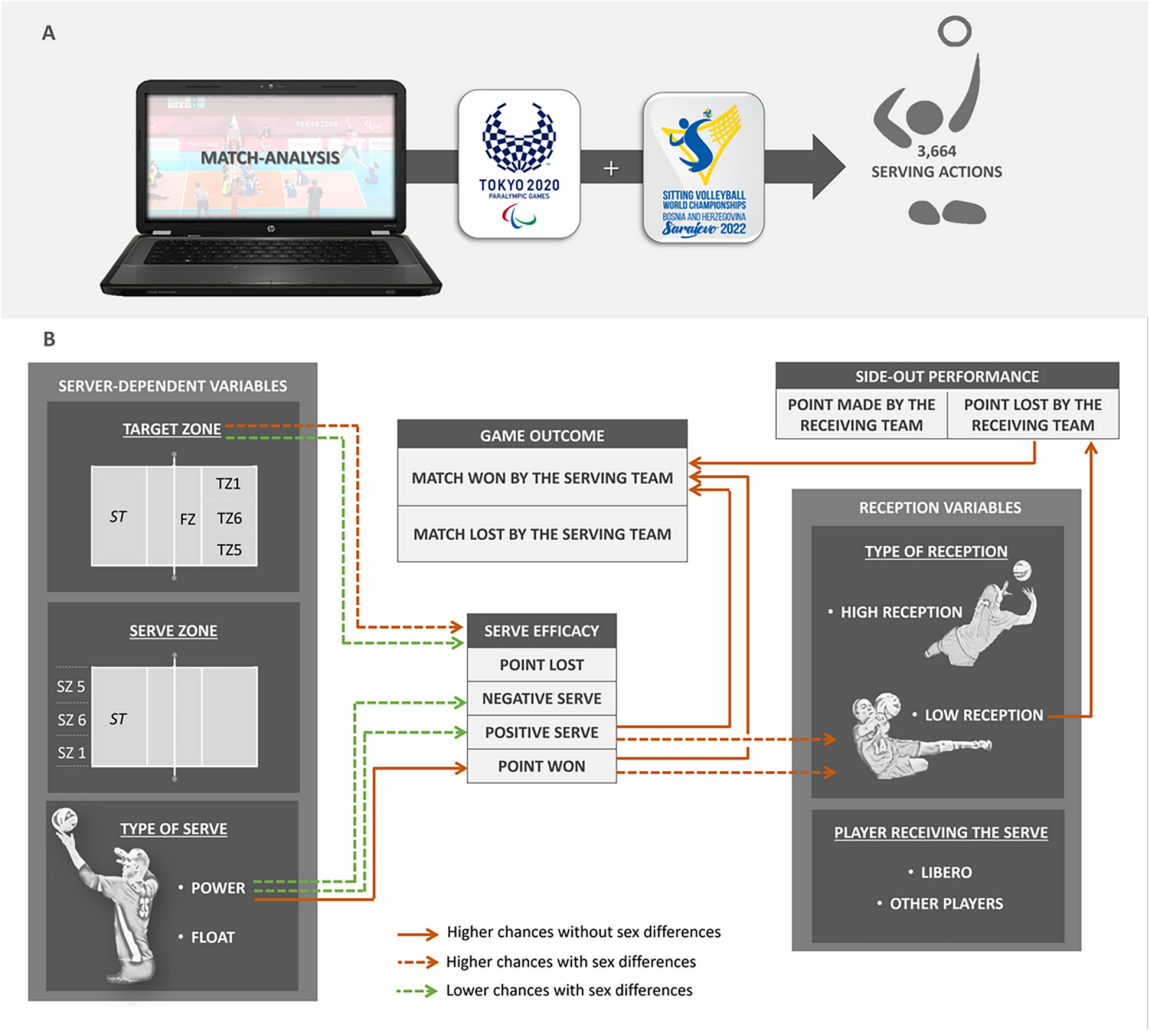
Panel **(A)**, graphical representation of the experimental setup; panel **(B)**, graphical summary of the results. ST, serving team's court; FZ, front zone; TZ1, Target Zone 1; TZ5, Target Zone 5; TZ6, Target Zone 6; SZ1, Serve Zone 1; SZ5, Serve Zone 5; SZ6 Serve Zone 6.

We applied a non-participative observational approach in line with the study's aims. As shown in Figure 1 (Panel A), the study sample comprised 3,664 serving actions, performed during the 2020 Tokyo Paralympic Games and the 2022 Sitting Volleyball World Championship held in Sarajevo. For both events, Championship was played according to the “Official Sitting Volleyball Rules 2017–2020” set out by World Paravolley under the surveillance of the International Paralympic Committee”.

The study was exempt from ethical approval because observing game actions poses no risk to the participants. All procedures performed in the study adhered to the Declaration of Helsinki and the ethical standards of the local ethics committee.

### 2.2 Data collection and handling procedures

The data were collected throughout the match analysis of the games played during the above-reported high-level international SV events. For each male and female event, we considered the following matches: gold final medal, bronze final medal, two semi-finals, 5th–6th place classification and 7th–8th place classification. Accordingly, we considered a total of 24 matches, 12 related to men's championship (6 from the 2020 Tokyo Paralympic Games and 6 from the 2022 Sitting Volleyball World Championship) and 12 related to women's championships (6 from the 2020 Tokyo Paralympic Games and 6 from the 2022 Sitting Volleyball World Championship). A total of eighty-five sets were analysed. The analysed game footages were free and available online from the following websites: World ParaVolley - YouTube and at the tokyo 2020 paralympic games sitting volleyball - YouTube.

For each match, data were annotated by an experienced observer who graduated in Sports Sciences with great experience as sport analyst in the Paralympic sports. The observer spent about three hours analysing each game. On any given day, a maximum of two games were analysed to reduce errors. All videos were re-observed by the operator, and another expert independently re-evaluated them to assess data reliability. The intra- and inter-observer Cohen's Kappa values for all considered variables were consistently above 0.75, the threshold indicating almost perfect agreement (26). A total of 3,664 serving actions were analysed (males: *n* = 1,910; females: *n* = 1,754). For each serving action, we considered the following variables: serve efficacy, serve zone, type of serve technique, target zone of the serve, type of reception, receiving player, and side-out performance (i.e., point won or point lost by the receiving team during the first action after the serve), and match outcome.

To assess efficacy, we adopted the categories used in the “Data Volley System, Data Project” (3, 8, 26), with adaptations specific to SV (e.g., the possibility for the receiving team to block or attack the serve). Serve efficacy was classified on a scale from 1 to 4 points, representing increasing levels of effectiveness: Point lost, Negative serve, Positive serve, and Point won. These categories help evaluate the effectiveness of serves and understand their impact on match outcomes.

In the Point lost category, we included serves resulting in a direct point scored against the serving team, including serve errors (e.g., the ball did not cross the net, went out of bounds, collided with an out-of-bounds item, or the server's buttocks lifted or touched the playing court during the serve) and blocked serves where the receiving team scored a point by directly blocking the serve. In the Negative serve category, we included serves associated with a perfect reception by the receiving team, allowing the setter all possible passing options. This category also includes serves blocked by the receiving team where the ball remained in their playing field, allowing them to attack. In the Positive serve category, we included serves associated with a reception by the opposing team that limited the setter's passing options (e.g., the setter cannot play the first tempo) or where the ball returned to the opposite court without being attacked. This category also includes serves blocked by the receiving team where the ball was returned directly to the serving court, allowing the serving team to counterattack. In the Point won category, we included aces and serves where the receiving team did not touch the ball or failed to return it.

The serve zone was defined as the area from which the serve was executed, covering a 6-meter wide space located behind the baseline and extending to the sidelines of the court. The serve zones, adapted from Quiroga et al. (27), included three specific areas: zone 1 (a 2-meter wide strip from the right sideline), zone 6 (a 2-meter wide strip located in the center, 2 meters from each sideline), and zone 5 (a 2-meter wide strip from the left sideline).

For the serve technique, we considered two types based on the ball's flight trajectory after striking: the float serve and the power serve. The float serve is characterized by minimal contact with the ball, resulting in a non-rotating, fluctuating, and unpredictable trajectory (26, 28). The power serve involves a strong, forward-rotating hit, imparting significant power and speed to the ball (26, 28).

The target zone of the serve was defined as the area in the opponent's court where the serve was received, categorized into four areas: front zone, zone 1, zone 6, and zone 5. The front zone, measuring 6 × 2 meters, is the area near the net demarcated by the center and attack lines. Zone 1 is a 2 × 3 meter area on the right side of the back zone. Zone 6 is a 2 × 3 meter area in the center of the back zone, and zone 5 is a 2 × 3 meter area on the left side of the back zone (27).

The type of reception, based on previous literature (23, 29), was categorized into high reception (overhand pass) and low reception (forearm pass).

We also evaluated the effectiveness of the side-out phase concerning the Negative serve and Positive serve categories (excluding Point lost and Point won categories). The side-out phase was defined as the first offensive action by the receiving team and categorized into errors made in side-out (an error by the receiving team allowing the serving team to score) and point won in side-out (the receiving team scoring directly in the side-out phase).

The match outcome was recorded as a dichotomous variable (won or lost match). In SV, a match is won when a team wins three sets. The first four sets are won by the team that first scores 25 points (with a minimum 2-point lead), while the fifth set is won by the team that first scores 15 points (with a minimum 2-point lead).

### 2.3 Statistical analysis

Data from match analysis typically exhibit a hierarchical structure, with individual match observations nested within players, who are further nested within sets, games, or teams. Mixed-effects regression models are particularly suited for this type of data because they account for variability at different levels of the hierarchy, capturing both within-group and between-group differences. By incorporating fixed effects to model overall trends (such as the average performance across groups of players) and random effects to capture individual or group-specific variations (such as differences in performance between individual players or teams), mixed-effects regression models provide a general and accurate analytical framework. To analyse the relationship between dependent binary variables (match outcome, type of reception, side-out performance) and independent variables (serve efficacy, type of reception, and side-out performance), we used two-level nested mixed-effects logistic models. Conversely, a two-level nested mixed-effects multinomial logistic model was employed to model serve efficacy as a function of serve type, target zone, and receiving player. To estimate the coefficients of these models, an adaptive Gauss-Hermite quadrature method with 7 quadrature points was used. The variance-covariance structure for the random effects in these models was defined such that each random effect has a distinct variance and all covariances are set to zero.

The assessment of gender-related differences in the relationship between an independent variable and a response variable was conducted using a likelihood-ratio (LR) test. This test compared the likelihood of the mixed-effect regression model that includes an interaction term (gender × independent variable) with the likelihood of the model that omits this interaction. If the interaction term proved to be statistically significant, it indicated that the association between the independent variable and the response variable differed between males and females. Consequently, the association was then estimated separately for each gender.

Statistical analyses were performed using Stata 18 (StataCorp. College Station, TX, USA) and R 4.3.2 (Foundation for Statistical Computing, Vienna). The statistical significance was set at *p*-value ≤ 0.05.

## 3 Results

### 3.1 Association between serve efficacy and match outcome

The results of the association analysis are detailed in Table 1. The adopted model showed that serve efficacy is significantly associated with the match outcome; increasing serve efficacy (e.g., moving from a Point lost serve to a Point won serve) was found to be associated with increased chances of winning the game. More precisely, the ratio between the probability of winning and losing the game (W/L ratio) starts at 0.86 for a Point lost and increases to 1.04, 1.48, and 1.67 for Negative serve, Positive serve, and Point won, respectively, with significant relative increments (*p* < 0.001) in the last two steps.

**Table 1:** Analysis of the association between match outcome (the dependent variable) and serve efficacy, side-out performance, and counterattack performance.

	Match outcome			Mixed-effects logistic regression		Gender interaction
	Lost	Won	W/L ratio	OR (95% CI)	P*	P**
Serve efficacy (*n*, %)						0.9
Point lost	198 (53.7)	171 (46.3)	0.86	Reference		
Negative serve	644 (49)	670 (51)	1.04	1.2 (0.86–1.66)	0.3	
Positive serve	650 (40.4)	961 (59.6)	1.48	1.71 (1.24–2.36)	0.001	
Point won	126 (37.5)	210 (62.5)	1.67	1.93 (1.42–2.63)	<0.001	
Total	1,618 (44.6)	2,012 (55.4)	1.24			

Percent relative frequencies are calculated by row.

P* = *p*-value of the Wald test used to assess the statistical significance of individual coefficients in the model.

P** = *p*-value of the likelihood ratio test used to assess the statistical significance of the (gender × covariate) interaction term.

### 3.2 Association between the considered server-dependent variables and the serve efficacy

The associations between the server-dependent variables and serve efficacy are detailed in Tables 2, 3. Our analysis found no statistically significant gender differences in the associations with serve zone (Table 2). Furthermore, no significant associations were observed between serve zone and serve efficacy. Significant gender interactions were observed in the associations of serve efficacy with both the type of serve and target zone (*p* < 0.001 and *p* = 0.024, respectively) (Table 3).

**Table 2:** Sample distribution (total and relative) of the server-dependent variables that did not show a statistically significant gender effect.

	Serve efficacy							Gender interaction
	Point lost (pl)	Negative serve (ns)	Positive serve (ps)	Point won (pw)	ns/pl ratio OR (95% CI)	ps/pl ratio OR (95% CI)	pw/pl ratio OR (95% CI)	*P*
Serve zone (*n*, %)								0.9
Zone 1	238 (10.4)	818 (35.7)	1,014 (44.2)	223 (9.7)	3.43 Reference	4.25 Reference	0.93 Reference	
Zone 6	54 (9.9)	203 (37.3)	236 (43.4)	51 (9.4)	3.77 1.22 (0.85–1.74)	4.38 1.07 (0.71 -1.63)	0.95 0.98 (0.61–1.59)	
Zone 5	77 (9.7)	292 (36.9)	361 (45.6)	62 (7.8)	3.80 1.09 (0.77–1.54)	4.70 1.1 (0.78–1.55)	0.80 0.86 (0.59–1.26)	
Total	369 (10.2)	1,313 (36.2)	1,611 (44.4)	336 (9.3)	3.55	4.35	0.91	
Receiving player (*n*, %)		Serve efficacy						Gender interaction
		Negative serve (ns)	Positive serve (ps)	Point won (pw)				*P*
						ps/ns ratio OR (95% CI)	pw/ns ratio OR (95% CI)	0.3
Other players		907 (40.1)	1,140 (50.4)	216 (9.5)		1.26 Reference	0.24 Reference	
Libero		290 (40.4)	358 (49.9)	69 (9.6)		1.24 0.99 (0.84–1.18)	0.24 1.01 (0.73–1.41)	
Total		1,197 (40.2)	1,498 (50.3)	285 (9.6)		1.25	0.24	

Analysis of the association between serve efficacy (the dependent variable) and serve zone and, serve direction. Percent relative frequencies are calculated by row. ORs (with 95% confidence intervals) were estimated using mixed-effect multinomial logistic regression.

*P* = *p*-value of the likelihood ratio test used to assess the statistical significance of the (gender × serve efficacy) interaction term.

**Table 3:** Sample distribution (total and relative) of the server-dependent variables that showed a statistically significant gender effect.

Male group	Serve efficacy							Gender interaction
	Point lost (pl)	Negative serve (ns)	Positive serve (ps)	Point won (pw)	ns/pl ratio OR (95% CI)	ps/pl ratio OR (95% CI)	pw/pl ratio OR (95% CI)	*P*
Type of serve (*n*, %)								<0.001
Float serve	72 (5)	647 (45.4)	637 (44.6)	72 (5)	9.08 Reference	8.92 Reference	1.00 Reference	
Power serve	87 (18.9)	139 (30.1)	172 (37.3)	63 (13.7)	1.59 0.17 (0.11–0.27)***	1.97 0.22 (0.13 -0.36)***	0.72 0.74 (0.43–1.27)	
Total	369 (10.2)	1,313 (36.2)	1,611 (44.4)	336 (9.3)	3.55	4.35	0.91	
Target zone								0.024
Zone 1	38 (6.4)	252 (42.3)	262 (43.9)	44 (7.4)	6.61 Reference	6.86 Reference	1.16 Reference	
Front zone	34 (13.8)	89 (36.2)	102 (41.5)	21 (8.5)	2.72 0.4 (0.2–0.83)*	3.01 0.44 (0.25–0.77)**	0.62 0.52 (0.25–1.08)	
Zone 5	21 (4.9)	186 (43)	194 (44.9)	31 (7.2)	8.78 1.35 (0.91–2.01)	9.16 1.35 (1.03–1.76)*	1.47 1.26 (0.62–2.57)	
Zone 6	19 (3.3)	264 (45.9)	253 (44)	39 (6.8)	13.91 2.11 (1.07–4.16)*	13.33 1.94 (1.12–3.35)*	2.06 1.77 (0.86–3.62)	
Total	112 (6.1)	791 (42.8)	811 (43.8)	135 (7.3)	7.02	7.18	1.20	
Female group	Serve efficacy							Gender interaction
	Point lost (pl)	Negative serve (ns)	Positive serve (ps)	Point won (pw)	ns/pl ratio OR (95% CI)	ps/pl ratio OR (95% CI)	pw/pl ratio OR (95% CI)	*P*
Type of serve (*n*, %)								<0.001
Float serve	125 (10)	387 (31)	595 (47.6)	142 (11.4)	3.10 Reference	4.76 Reference	1.14 Reference	
Power serve	85 (17.6)	136 (28.1)	204 (42.2)	59 (12.2)	1.60 0.57 (0.41–0.79)**	2.40 0.5 (0.36–0.69)***	0.69 0.6 (0.39–0.91)*	
Total	210 (12.1)	523 (30.2)	799 (46.1)	201 (11.6)	2.50	3.81	0.96	
Target zone								0.024
Zone 1	52 (10.4)	128 (25.5)	255 (50.8)	67 (13.4)	2.45 Reference	4.88 Reference	1.29 Reference	
Front zone	57 (36.1)	46 (29.1)	37 (23.4)	18 (11.4)	0.81 0.33 (0.18–0.62)***	0.65 0.13 (0.08–0.22)***	0.32 0.24 (0.13–0.46)***	
Zone 5	26 (7)	116 (31)	184 (49.2)	48 (12.8)	4.43 1.75 (0.93–3.30)	7.03 1.45 (0.87–2.43)	1.83 1.44 (0.84–2.49)	
Zone 6	37 (5.6)	184 (49.2)	322 (48.9)	68 (10.3)	8.79 2.61 (1.64–4.15)***	8.73 1.77 (1.11–2.83)*	1.84 1.42 (0.83–2.43)	
Total	172 (10.2)	522 (30.8)	798 (47.1)	201 (11.9)	3.02	4.62	1.17	

Analysis of the association between serve efficacy (the dependent variable) and type of serve and target zone for males and females separately. Percent relative frequencies are calculated by row. ORs (with 95% confidence intervals) were estimated using mixed-effect multinomial logistic regression.

Asterisks placed close to the OR confidence intervals indicate the *p*-value of the Wald test used to assess the statistical significance of individual coefficients in the model: * for *p* < 0.05, ** for *p* < 0.01, and *** for *p* < 0.001.

*P* = *p*-value of the likelihood ratio test used to assess the statistical significance of the (gender × serve efficacy) interaction term.

In males, compared to the float serve, the power serve was associated with a higher probability of losing the point (from 5% to 18.9%), and lower chances of obtaining a Negative serve (from 45.4% to 30.1%) and a Positive serve (from 44.6% to 37.3%). The ratio between, respectively, negative serve and positive serve over point lost (ns/pl ratio and ps/pl ratio) showed a statistically significant decrease by 83% (ns/pl ratio from 9.08 to 1.59) and by 78% (ps/pl ratio from 8.92 to 1.97). Analogously, in females, the power serve was associated to negative serve, positive serve and point won, with decreased chances of all of them with respect to point lost. In fact, the corresponding ns/pl ratio, ps/pl ratio and pw/pl ratio moved respectively from 3.10, 4.76 and 1.14 to 1.60, 2.40, and 0.69, with statistically significant decreases of 43%, 50%, and 40% (Table 3). The reader may wonder how an apparent increase in the probability of point won (from 11.4% to 12.2%) can be consistent with an interpretation in the sense of decreased chances of winning the point with respect to losing it. The answer lies in the fact that the point lost is the reference category, so the probability of point won is not considered absolute, but with respect to that of the point lost: in this respect, the slight increase in the probability of a point won (from 11.4% to 12.2%, as mentioned above) is not enough to compensate the substantial increase in the likelihood of a point lost (from 10% to 17.6%), so the comparison between the two situations is any way in favour of the decreased chance of point won with respect to point lost.

In males (Table 3, top panel), the investigation of the relationship between the target zone and serve efficacy revealed that, compared to zone 1, directing the ball to the front zone is associated with an increased probability of losing a point by serving (from 6.4% to 13.8%), along with reduced chances of achieving both a Negative serve (from 42.3% to 36.2%) and a Positive serve (from 43.9% to 41.5%) with respect to a point lost. The corresponding ns/pl ratio and ps/pl ratio moved from 6.61 to 2.72 and from 6.86 to 3.01, respectively, with statistically significant decreases of 60% and 56%. The opposite occurs when directing the ball to zone 6, rather than zone 1. This choice determines appreciably higher ns/pl ratio and ps/pl ratio (from 6.61 to 13.91 and from 6.86 to 13.33) that exhibited statistically significant increments of 111% and 94%. At first glance, the modest improvements in the probability of a negative serve (from 42.3% to 45.9%) and a positive serve (from 43.9% to 44%) might appear surprising given the substantial increases in the ns/pl and ps/pl ratios. However, as before, this evidence can be understood by considering the presence of a reference category: the corresponding high reduction in the probability of point lost (the reference category, halving from 6.4% to 3.3%), enhances the (seemingly) modest reductions in the likelihoods of negative serve and positive serve. Again, the point is that the chances of negative serve and positive serve did not increase in absolute terms, but with respect to the chance of point lost.

In females (Table 3, bottom panel), the analysis showed that, compared to zone 1, directing the ball to the front zone is associated with an increased probability of point lost a from 10.4% to 36.1%, which determines statistically significant decreases in ns/pl ratio, ps/pl ratio and pw/pl ratio of, respectively 67%, 87% and 76% (from 2.45, 4.88 and 1.29 to 0.81, 0.65 and 0.32). Similarly to what evidenced above, the reader should not be confused by the apparent increase in the probability of negative serve (from 25.5% to 29.1%), which instead should be considered as lower chances of negative service with respect to point lost, as its slight increase is not enough to compensate the strong increase in the probability of the latter (from 10.4% to 36.1%, as mentioned above). Serving in zone 6 rather than to zone 1, almost halves the likelihoods of point lost (from 10.4% to 5.6%), with a corresponding increase in pn/pl ratio and ps/pl ratio from 2.45 to 8.79 and from 4.88 to 8.73 (statistically significant increments of 161% and 77%).

### 3.3 Association between serve efficacy and type of reception, between type of reception and side-out performance, and between side-out performance and match outcome

When investigating the relationship between serve efficacy (categorized as Negative Serve, Positive Serve, and Point Won) and the quality of reception, a significant gender × quality interaction was observed (*p* < 0.001) (Table 4).

**Table 4:** Analysis of the association between serve efficacy and type of reception.

Male group	Type of reception (*n*, %)			Mixed-effects logistic regression		Gender interaction
	High	Low	Low/High ratio	OR (95% CI)	P*	P**
Serve efficacy						<0.001
Negative serve	662 (92.6)	53 (7.4)	0.08	Reference		
Positive serve	609 (84)	116 (16)	0.19	2.43 (1.94–3.04)	<0.001	
Point won	56 (63.6)	32 (36.4)	0.57	8.13 (3.31–19.9)	<0.001	
Total	1,327 (86.8)	201 (13.2)	0.15			
Female group	Type of reception (*n*, %)			Mixed-effects logistic regression		
	High	Low	Low/High ratio	OR (95% CI)	P*	
Serve efficacy						<0.001
Negative serve	303 (62.9)	179 (37.1)	0.59	Reference		
Positive serve	390 (50.6)	381 (49.4)	0.98	1.57 (1.21–2.03)	0.001	
Point won	77 (44.5)	96 (55.5)	1.24	1.98 (1.42–2.76)	<0.001	
Total	770 (54)	656 (46)	0.85			
	Side-out performance (*n*, %)			Mixed-effects logistic regression		Gender interaction
	Point	Error	Error/Point ratio	OR (95% CI)		
Type of reception						0.2
High	593 (54.9)	487 (45.1)	0.82	Reference		
Low	177 (41.7)	248 (58.3)	1.40	1.68 (1.24–2.29)	<0.001	
Total	770 (51.2)	735 (48.8)	0.95			

P* = *p*-value of the Wald test used to assess the statistical significance of individual coefficients in the model.

P** = *p*-value of the likelihood ratio test used to assess the statistical significance of the (gender × covariate) interaction term.

For male players, the percentage of low reception was 7.4% for Negative Serve, 16% for Positive Serve, and 36.4% for Point Won, with corresponding Low/High probability ratios of 0.08, 0.19, and 0.57, respectively. The increase in the Low/High ratio for Positive Serve and Point Won serves, compared to Negative Serve, was 238% and 714%, respectively (*p* < 0.001). For female players, the percentages of low reception for Negative, Positive, and Point Won serves were 37.1%, 49.4%, and 55.5%, respectively. The Low/High ratio increased by 165% and 210% for Positive and Point Won serves, compared to Negative Serve, with statistical significance (*p* = 0.001 and *p* < 0.001, respectively).

When considering the association between the type of reception and performance in the side-out phases, the analysis revealed that, compared to a high reception (the reference group), a low reception was associated with a higher chance of the receiving team committing an error in the side-out phase (probability from 45.1% to 58.4%), with the ratio between the probability of error and point increasing from 0.82 to 1.40 (+171%) (Table 4).

The analysis indicated that an error in the side out phase by the receiving team significantly increased the probability of the serving team winning the game from 45.7% to 61.4%, with a W/L ratio moving from 0.84 to 1.59 (a statistically significant increment of 89%, *p* < 0.001) (Table 5).

**Table 5:** Analysis of the association between match outcome (the dependent variable) and side-out performance.

	Match outcome			Mixed-effects logistic regression		Gender interaction
	Lost	Won	W/L ratio	OR (95% CI)	P*	P**
Side-out performance (*n*, %)						0.5
Side-out point	482 (54.3)	405 (45.7)	0.84	Reference		
Side-out error	335 (38.6)	533 (61.4)	1.59	1.89 (1.56–2.3)	<0.001	
Total	817 (46.5)	938 (53.6)	1.15			

Percent relative frequencies are calculated by row.

P* = *p*-value of the Wald test used to assess the statistical significance of individual coefficients in the model.

P** = *p*-value of the likelihood ratio test used to assess the statistical significance of the (gender × covariate) interaction term.

## 4 Discussion

To date, little research has been conducted on SV game performance (1, 30, 31), and no data have been provided regarding the key elements associated with match outcomes. Using data collected from high-level male and female SV players in a real practice setting (i.e., World Championships and Paralympic games), this study first investigated the interrelated serve-reception task by exploring the association between the serve efficacy and the match outcome, the factors that may influence the serve efficacy, as well as the influence of the serve efficacy on both the reception task and the performance in the side-out phase. A graphical summary of the results is depicted in Figure 1 (Panel B). In agreement with the underlying hypothesis of our study and in line with previous findings on standing volleyball (6, 12, 14, 18, 32), our results indicated that serve efficacy in SV, which refers to the performance or effect achieved with the serve, is significantly associated with match outcomes. Specifically, we observed that increased serve efficacy was associated with higher chances of winning the game. This positive trend provides evidence of the importance of performing a serve that allows the serving team to score a direct point or that puts the opposing team in trouble in constructing their attacking action. More specifically, in line with standing volleyball (6), it has been shown that serves included in both the Positive serve and Point won categories gave the serving team statistically significantly higher chances to win the game. Positive serves, namely the serves that did not allow the receiving team the possibility to attack the ball with all the attacking options, increased by 46.3% to 59.6% the chance to win the game, and the Point won serves, namely the serves where the receiving team does not touch the ball or fails to return it, increased by 46.3% to 62.5% the probability of winning. On the other hand, service errors were associated with a reduced chance of obtaining a positive game outcome, while a Negative serve, namely a serve associated with a perfect reception by the receiving team, was not associated either with a higher chance of winning or a higher chance of losing the match. Achieving a direct point through the service or the ability of the service to impair directly or indirectly the development of their first attacking action by the opposing team is even more important than service error management and it is directly related to the positive final outcome of the competition.

Of note, the results did not show any statistically significant gender effect on the association between serve efficacy and the match outcome. Consistent with standing volleyball literature (12, 33), since the serve can be considered a terminal action that may result in a direct point, it would be therefore important for SV players of both genders to increase its efficacy. Based on these results, we can infer that the serve is crucial to the performance of both male and female SV teams and training programs aimed at mastering this skill are of vital importance in SV to increase the chance of winning a match.

Understanding the factors that determine serve efficacy is crucial for developing a winning serve. Therefore, a secondary aim of this study was to evaluate the variables that influence serve efficacy in high-level male and female SV players. In SV, the serve is a closed-skill task where the player has full control over several variables (i.e., server-dependent variables). Among the server-dependent variables considered (i.e., serve zone, target zone, and type of serve), the results revealed that the serve zone was not associated with serve efficacy in either males or females. This result aligns with previous literature on standing volleyball athletes (34, 35) and suggests that the player's position on the court while serving does not influence the efficacy of the serve in either gender.

It is interesting to report that the both the target zone and the type of serve were associated with serve efficacy, but the extent of such associations differ between male and female. As far as the type of serve is concerned (i.e., power or float serve), the results underlined that both in males and females the power serve was associated to a higher chance of scoring a direct point but also to a higher probability of making an error and, consequently, losing the point. It is important to bear in mind that, as compared to the float serve, the power serve has a greater power and speed thereby reducing the receiver reaction time and minimizing the opponent's attack options. In line with data about standing volleyball (27), in SV the use of power serves increased the serve risk while attempting to hinder the organization of the opponent attack with the goal of scoring points in both males and females. However, it is interesting to note that the chances of making an error while serving and accordingly losing the point are higher than the chances of scoring a direct point, especially in females. This result should raise a question as to whether or not to force the serve to try to score a direct point, in the face of a higher probability of making an error. Future research is needed to better understand this aspect also by the possible influence of some situational variables, like match status, match period, score and quality of opposition.

When considering the target zone, the results showed that directing the ball to the front zone near the net is associated with an increased probability of the serving team losing the point compared to Zone 1. This increased probability can be attributed to the strategic advantages it gives the receiving team, such as setting up effective attacks and challenging the serving team's defense and blocking. Therefore, coaches and players should aim to minimize serves directed to the front zone and avoid having them blocked by the opponents. Instead, they should focus on directing the ball away from the net and the opponents' block to increase pressure on the receiving team. When interpreting this result, it is important to consider that in SV, the serve can be directly blocked by the opposing team. In this study, blocked serves were categorized as falling into the front zone. Future research could explore blocked serves in greater detail, distinguishing between those where the play ends after the block and those where the action continues. Additionally, it would be valuable to examine serves that are not blocked separately, to better understand the specific characteristics of SV, particularly regarding the zones targeted by players and their association with serve efficacy. A third aspect assessed in this study was the relationship between serve efficacy and reception (i.e., the type of reception and the role of the receiving player), and how the type of reception affected side-out performance. The results confirmed the hypothesis, showing that serve efficacy influences the reception task. This result aligns with previous findings in standing volleyball, which report that serve technique significantly influences reception efficacy, with jump serves increasing the number of receptions that do not facilitate an opponent's attack (28). Specifically, the results of the present study showed that, compared to Negative serves, Positive serves were associated with a higher probability that the opponent team would use a low reception (i.e., forearm pass) rather than a high reception (i.e., overhand pass). Similar to the literature on standing volleyball (23, 35, 36), it can be argued that in the SV game, a low reception is less accurate than a high reception, thereby reducing the receiving team's ability to build an efficient attack. Conversely, a high reception provides the receiving team with better opportunities to construct their attack.

A relationship between serve efficacy and the type of reception was expected because receivers rely on the kinematics of the server and the serve itself to gather information for their reception and subsequent passing (19). From an ecological dynamics perspective (37), both the constraints of the ball's approach and those of the receiving player will determine how the serve is handled (i.e., type of pass used) and, consequently, the effectiveness of the pass to the setter and the potential to execute a successful attack.

The results also showed no statistically significant associations between serve efficacy and the receiving player (i.e., whether the ball was received by the libero or other players) (Table 2). Before interpreting these results, it is important to remember that in standing volleyball, the libero player was introduced in 1998 to counterbalance attack supremacy over defense. The libero is a defensive specialist with expertise in service reception and defense. Consequently, receptions were the actions most frequently performed by the libero player in any game phase, including free-ball situations (38). Moreover, it has been demonstrated in standing volleyball that when the libero performs in defense phases, reception performance improves and attack efficacy increases (39).

In analogy with standing volleyball, we would have expected the libero to handle serves more effectively, providing accurate and controlled passes to the setter. Surprisingly, our data showed that the libero did not receive the majority of serves and did not handle serves more effectively compared to other players. This suggests that in SV, the role of the libero might be less specialized in reception than in standing volleyball. The dynamics of SV might differ slightly due to the nature of the game, as players move on the court in a seated position. Therefore, the serving team may target other players to avoid the libero, as their superior skills could lead to a better offensive setup for the receiving team.

Another interesting consideration is that in SV, the physical characteristics of athletes are less pronounced than in standing volleyball. In SV, an athlete's stature or agility is primarily affected by the type and severity of their impairment rather than their innate characteristics.

The aforementioned considerations were supported by our observation that a low reception was associated with a higher probability of the receiving team making an error in the side-out phase. Notably, when the receiving team made an error in this phase, the serving team's probability of winning the game increased by approximately 61.4% compared to when the receiving team scored a point. This result was consistent across both genders, suggesting that forcing the receiving team into errors during the side-out phase through effective serves increases the chances of winning in both male and female games. Furthermore, consistent with the literature (23, 40), these findings highlight that performance in the side-out phase is crucial for competitive success in both male and female volleyball.

This study has some limitations that should be considered. First, although the study was limited to the best 8 teams in each championship, the quality of the opponents was not taken into account. Second, we were unable to consider the impact of the type and severity of the athletes' impairments on the variables studied. Future research should also examine whether certain types of impairment (e.g., upper limb impairments) have a different impact on serve efficacy compared to others (e.g., lower limb impairments).

A notable strength of this study was the statistical methods used to explore the associations between variables. Traditional statistical methods in sport sciences often rely on generalized linear models, which are not suitable for analysing match data because they ignore the hierarchical structure of the data. In contrast, the mixed-effects models employed in this study, which consider both fixed and random effects, provide a comprehensive analytical framework for modelling overall trends as well as individual or group-specific variations.

In conclusion, the results presented in this study provide an initial step toward describing serve performance in high-level male and female SV competitions. These findings should be considered in the training processes for high-level male and female SV players. Based on these results, it is recommended that high-level coaches focus on improving their players' serve efficacy by instructing them on managing risk, avoiding serves to the front zone, and reducing the likelihood of overhead receptions.

Since serve performance involves continuous interaction between the ball, the server, and the receiver (41), it is essential to gather more detailed information about the variables that most effectively explain serve performance in various competitive settings. Accordingly, future research in this Paralympic sport should build on these findings to explore the complex interactions of various variables involved in SV games that could affect competition outcomes. These variables may include technical, tactical, and strategic factors, as well as aspects related to the physical characteristics of individual athletes, such as their anthropometric traits, strength levels, types and severity of impairments, and emotional management during competition. It is important to further investigate these factors to analyse their impact on match outcomes and the various stages of competition. Additionally, research aimed at establishing performance profiles for different types of sets and competition categories would help develop optimal serve strategies. In this regard, statistical and machine learning methods, such as clustering and principal component analysis, could be employed for these purposes. These approaches have been applied in standing team sports such as soccer, basketball, and volleyball (41–46), as well as in Paralympic sports like wheelchair basketball (47). This would assist coaches and technical staff in creating effective training strategies based on players' competitive levels, allowing them to set coherent objectives aligned with competition demands and design tasks consistent with these objectives. Coaches and practitioners can use this data as a reference for SV players to anticipate match outcomes and identify key areas for improvement during practice to achieve better technical performance.

## Data Availability

The raw data supporting the conclusions of this article will be made available by the authors, without undue reservation.
